# Distribution of urinary gamma-glutamyltransferase activity in 40- to 74-year-old Japanese women

**DOI:** 10.1016/j.plabm.2020.e00161

**Published:** 2020-04-05

**Authors:** Akihisa Hata, Maki Miyauchi, Yoshio Suzuki, Yuki Otomo, Noboru Fujitani

**Affiliations:** a Faculty of Veterinary Medicine, Okayama University of Science, Ikoino-oka 1-3, Imabari, Ehime, 7948555, Japan; b Graduate School of Health and Sports Science, Juntendo University, Hiraga-gakuendai 1-1, Inzai, Chiba, 2701695, Japan; c Graduate School of Risk and Crisis Management, Chiba Institute of Science, Shiomi-cho 15-9, Choshi, Chiba, 2880025, Japan; d Biomedical Science Examination and Research Center, Okayama University of Science, Ikoino-oka 1-3, Imabari, Ehime, 7948555, Japan

## Abstract

Urinary gamma-glutamyltransferase (u-γGT) concentration (U/L) and excretion (urinary creatinine-corrected u-γGT; u-γGT/u-Cre, U/g creatinine) are useful markers for kidney disease. However, there is limited information available on u-γGT and u-γGT/u-Cre distribution in the elderly Japanese population. In this study, we investigated the distribution of u-γGT and u-γGT/u-Cre in 113 Japanese women aged 40–74 years. The u-γGT was assessed from spot urine samples (collected from 09:00 to 14:00) spectrophotometrically according to the Japan Society of Clinical Chemistry reference measurement procedure using l-γ-glutamyl-3-carboxy-4-nitroanilide as the substrate. The u-Cre was measured enzymatically using creatininase, creatinase, sarcosine oxidase, and peroxidase. None of the participants was diagnosed with any kidney disease. Median u-γGT and u-γGT/u-Cre values (central 95% interval values) were 29.7 (5.3–144.0) U/L and 57.9 (32.9–122.7) U/g creatinine, respectively. The distribution of u-γGT tended to decline with age. There was a statistically significant difference in the u-γGT value between the 40-59- and 60-74-year-old groups. In contrast, there was no significant difference in the u-γGT/u-Cre between each age group. The u-Cre level also declined with age. It is suggested that the decline of u-γGT with aging would be masked by the u-Cre correction.

## Highlights

• Median (central 95% interval values) u-γGT value was 29.7 (5.3–144.0) U/L.
• Median (central 95% interval values) u-γGT/u-Cre ratio was 57.9 (32.9–122.7) U/g creatinine.
• Distribution of u-γGT tends to decline with age.
• u-Cre levels also declined with age.
• Decline of u-γGT with aging would be masked by the u-Cre correction.

## 1 Introduction

Gamma-glutamyltransferase (γGT) is a membrane enzyme that plays a vital role in glutathione metabolism and shows particularly high activity in the kidney, pancreas, and liver [1,2]. In the kidney, γGT is strongly expressed on the luminal surface of the proximal tubular epithelial cells [3] and then excreted through the urine. Previous studies have indicated that the urinary γGT (u-γGT) concentration and/or excretion, as determined by the urinary creatinine-corrected value of u-γGT (u-γGT/u-Cre), can serve as biomarkers for proximal tubular injury given their increased levels in diseases and conditions, such as acute renal injury [4,5], systemic lupus erythematosus [6], heavy metal intoxication [7], drug intoxication [8,9], multiple myeloma [10], and failure of renal transplantation [11].

For practical applications as a biomarker, it is crucial to determine the u-γGT and u-γGT/u-Cre distribution in individuals without renal disease as a reference; however, this information is currently limited. In particular, there is limited information available on the u-γGT and u-γGT/u-Cre distribution in old Japanese adults. Given that u-γGT undergoes a physiological change during aging [12] and the urinary Cre concentration differs among age groups [13], it is necessary to establish the u-γGT and u-γGT/u-Cre distribution for each age group.

In Japan, “specific health checkups and specific health guidance” are carried out for all (40–74)-year-old insured individuals and their dependents to improve screening and detection of metabolic syndrome. Therefore, in this study, we used the data available from these “specific health checkups” to determine the distribution of γGT and u-γGT/u-Cre in (40–74)-year-old Japanese women. We then compared the u-γGT concentration and excretion levels in each age group within this cohort.

## 2 Materials and methods

### 2.1 Participants

Urine samples were obtained from (40–74)-year-old women (n ​= ​152) who received specific health checkups in Choshi, Japan in July and August 2017. Only women were included in the study since the primary participants of the health checkups offered by the local government carries are housewives and elderly individuals.

None of the women was hospitalized or resided in a nursing home at the time of the examination. Individuals were not excluded if they had an illness or disease history, as it is challenging to gather data from only completely physically healthy individuals, especially for more elderly individuals. However, given the likely influence of u-γGT on kidney disease, women diagnosed with kidney disease were excluded from the study. In addition, 37 people who showed eGFR <60 mL/min/1.73 ​m^2^ and/or urinary Alb ≥30 mg/g creatinine, which are two diagnostic criteria of chronic kidney disease in Japan [14], were excluded from the analysis. In total, urinary u-γGT was investigated in 113 (40–74)-year-old females (16 in their 40s, 23 in their 70s).

### 2.2 Sample and data collection

The urine samples were spot-urine collected from 9:00 to 14:00 and stored at 4 ​°C in sterilized polypropylene tubes; u-γGT analysis was carried out on the same day of collection as u-γGT activity is unstable [15]. Afterward, the urine samples were stored at −30 ​°C until u-Cre and u-albumin analyses. Collected urine samples were centrifuged at 1500×*g* for 10 ​min, and the supernatants were used for the analyses. Physical exam (height, weight, abdominal girth, systolic, and diastolic blood pressure) and biochemical blood parameter values [high-density lipoprotein-cholesterol (HDL-C), low-density lipoprotein-cholesterol (LDL-C), triglyceride (TG), asparagine aminotransferase (AST), alanine aminotransferase (ALT), serum-γGT (s-γGT), hemoglobin A1c (HbA1c; NGSP value), serum-creatinine (s-Cre), estimated glomerular filtration rate (eGFR; estimated by the eGFR equation for Japanese population [16])] were obtained from the Health and Welfare Center of Choshi.

### 2.3 Analysis of the urinary levels of γGT, Cre, and albumin

The u-γGT was measured according to the Japan Society of Clinical Chemistry (JSCC) reference measurement procedure using l-γ-glutamyl-3-carboxy-4-nitroanilide (GluCANA) as the substrate [17]. In brief, 0.15 ​mL of urine was dispensed into a test tube to which 2 ​mL of 159 ​mmol/L glycylglycine buffer (pH 7.90 ​at 30 ​°C) was added, and the mixture was incubated in a thermostat chamber at 37 ​°C for 5 ​min. Subsequently, 0.5 ​mL of 31.8 ​mmol/L GluCANA-glycylglycine buffer was added, and the mixture was further incubated at 37 ​°C for 1 ​min. Immediately after incubation, absorbance at 410 ​nm was measured at 37 ​°C for 3 ​min on a Hitachi 7012 model clinical spectrophotometer (Hitachi High-Technologies Corp., Tokyo, Japan). The apparent molar absorption coefficient (*app*.ε) of 5-amino-2-nitrobenzoate (5-ANB), used as the enzyme reaction indicator of our spectrophotometer, was 7525 ​L·mol^-^1·cm^-^1 (37 ​°C). The γGT activity was then calculated using the following formula:

$$\text{γGT ​}\left(U/L\right)=\frac{\text{ΔAbs}/\text{min}}{app.\text{ε}}\times \frac{V}{v}\times 10^{6}$$

where ΔAsb/min is the absorbance change per minute, *app. ε* is the apparent molar absorption coefficient (L·mol^-^1·cm^-^1), *V* is the total volume of the reaction solution (mL), and *v* is the sample volume (mL). In this analytical method, the lower and upper limits of quantitation were 2.3 and 800 U/L, respectively. The inter- and intra-assay coefficient of variations (CVs) were 2.15% and 1.71%, respectively.

Urinary creatinine (u-Cre) levels were also measured for the creatinine correction of the u-γGT by an enzymatic method using creatininase, creatinase, sarcosine oxidase, and peroxidase (L-type WAKO CRE・M, FUJIFILM Wako Pure Chemical, Osaka, Japan) and the clinical autoanalyzer, HITACHI 3100 (Hitachi High-Technologies Corp.). The urine sample was diluted 10-fold with ultrapure water. Controls WAKO-I and –II (FUJIFILM Wako Pure Chemical) were used for γGT and creatinine measurement quality control. Urinary albumin (u-Alb) levels were also measured by the immune nephelometric method using a polyclonal anti-human albumin antibody (Auto WAKO microalbumin, FUJIFILM Wako Pure Chemical) and the clinical autoanalyzer, HITACHI 3100. Control for Auto WAKO microalbumin (FUJIFILM Wako Pure Chemical) was used for u-Alb measurement quality control.

### 2.4 Statistical analysis

The normality of each clinical test value was checked by histogram and tested using the Shapiro-Wilk test. Parametric data were indicated as the mean ​± ​standard deviation, and nonparametric data were indicated as median and interquartile range. Correlations between u-γGT, u-Cre, u-γGT/u-Cre, and each medical examination variable were determined by Spearman correlation coefficients using subject means. Statistical significance was determined by a two-sided Mann-Whitney *U* test. Differences with a *P-*value *(P)* ​< ​0.05 were considered statistically significant. SPSS (version 19, IBM Japan, Tokyo, Japan) was used for statistical analyses.

## 3 Results

### 3.1 Distribution of u-γGT and u-γGT/u-Cre

**Fig. 1:**
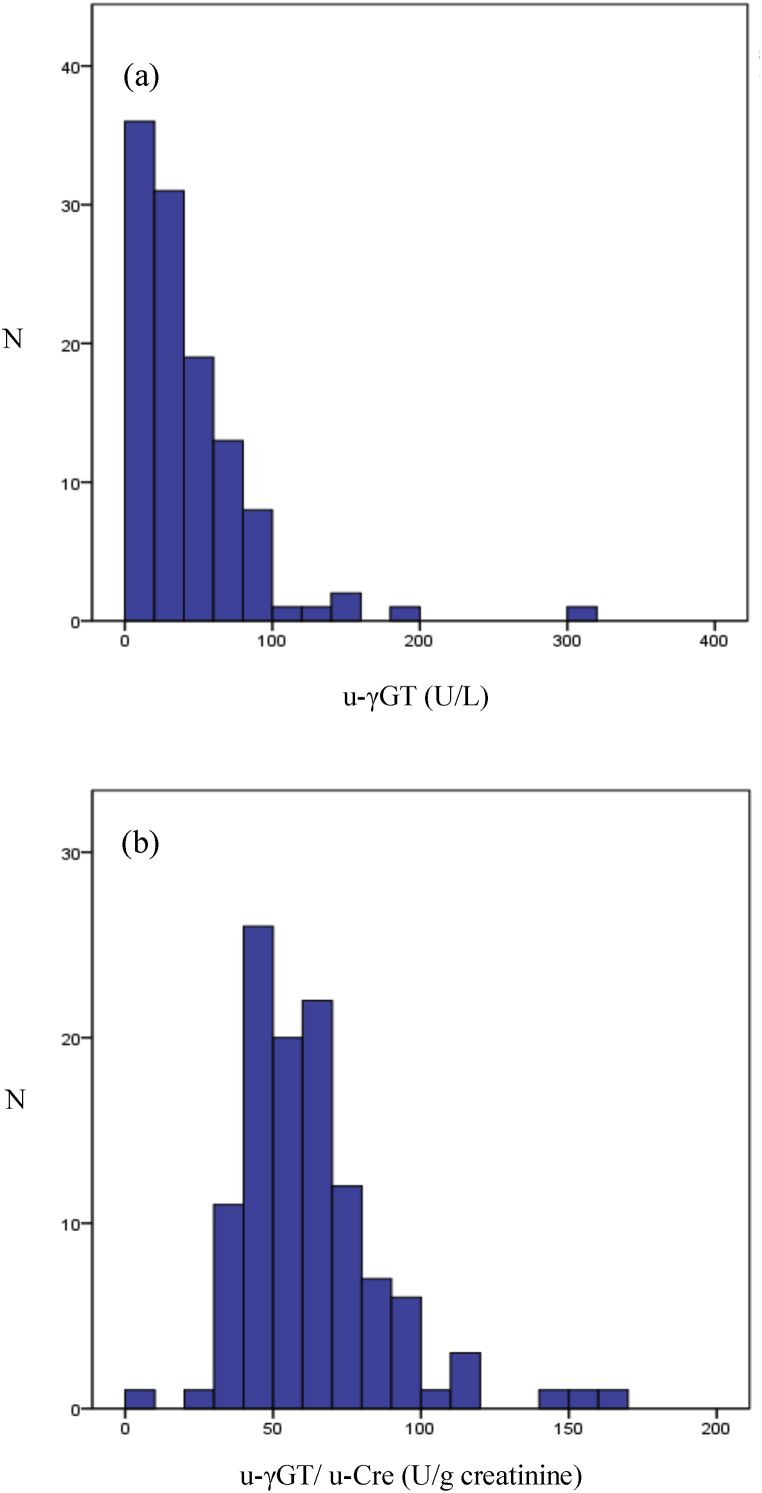
Histograms of the (a) u-γGT and (b) u-γGT/u-Cre levels in the 113 participants.

The fundamental statistics of physical exams, and blood and urinary biochemical parameter levels for the 113 participants are shown in Table 1. The histograms of the u-γGT and u-γGT/u-Cre values for the 113 participants are shown in Fig. 1. Both histograms display a left-skewed distribution. These distributions were indicated as non-parametric by the Shapiro-Wilk test. Therefore, values of u-γGT and u-γGT/u-Cre are shown as minimum and maximum, and 2.5, 25, 50 (median), 75, and 97.5 percentiles (Table 2). The u-γGT and u-γGT/u-Cre levels ranged from 3.1 to 302.5 U/L and from 8.0 to 168.1 U/g creatinine, respectively, in all the 113 participants.

**Table 1:** Physical exam and biochemical parameter values of the study subjects. Height and blood pressure are shown as mean ​± ​SD. Others are shown as median (interquartile range).

Variable		n ​= ​113
**Physical exam**		
Age (y)		66 (58–69)
Height (cm)		153 ​± ​6
Weight (kg)		50 (45–57)
Belly girth (cm)		80 (73–85)
Body mass index (kg/m^2^)		22 (19–24)
Systolic blood pressure (mmHg)		126 ​± ​16
Diastolic blood pressure (mmHg)		72 ​± ​10
**Blood biochemical parameter**		
HDL cholesterol (mmol/L)		1.6 (1.4–1.9)
LDL cholesterol (mmol/L)		3.1 (2.5–3.6)
Triglycerides (mmol/L)		1.0 (0.7–1.5)
AST (U/L)		20 (18–23)
ALT (U/L)		16 (13–19)
γGT (U/L)		17 (14–23)
HbA1c (NGSP value; %)		5.7 (5.5–6.0)
Creatinine (μmol/L)		56.6 (52.2–61.0)
eGFR (mL/min/1.73m^2^)		72.3 (66.1–79.2)
**Urine biochemical parameter**		
Albumin (mg/L)		2.6 (0.9–5.2)
Albumin/creatinine (mg/g creatinine)		5.1 (2.1–8.5)
Creatinine (g/L)	All subjects	0.58 (0.27–0.90)
	40s	0.85 (0.32–1.44)
	50s	0.74 (0.52–1.05)
	60s	0.49 (0.24–0.89)
	70s	0.56 (0.30–0.72)

**Table 2:** Urinary γGT and γGT/creatinine values of the study subjects.

	All subjects	40s	50s	60s	70s
N	113	16	14	60	23
γGT (U/L)					
Minimum	3.1	7.9	18.0	3.1	4.3
2.5 percentile	5.3	8.1	18.4	5.6	4.5
25 percentile	17.0	20.1	31.8	14.6	14.9
Median	29.7	45.7	46.9	25.0	27.4
75 percentile	54.2	91.0	61.2	54.2	42.5
97.5 percentile	144.0	262.6	95.0	115.2	73.0
Maximum	302.5	302.5	108.4	143.0	78.2
γGT/creatinine (U/g creatinine)					
Minimum	8.0	43.3	41.4	8.0	35.0
2.5 percentile	32.9	43.5	42.2	29.6	35.4
25 percentile	45.6	53.5	46.9	44.8	41.0
Median	57.9	60.6	59.1	59.4	51.7
75 percentile	74.9	78.0	70.4	77.2	66.1
97.5 percentile	122.7	139.0	90.3	127.2	98.0
Maximum	168.1	151.7	90.9	168.1	107.7

The distributions of u-γGT and u-γGT/u-Cre tended to decline with age. The u-γGT drastically decreased between the 50s and 60s. There was a statistically significant difference in the u-γGT value between the (40–59)- and (60–74)-years-old groups according to the Mann-Whitney *U* test (*P* ​= ​0.007). In contrast, the u-γGT/u-Cre showed a slight decrease between the 60s and 70s, whereas the difference in u-γGT/u-Cre values between (40–69)- and (70–74)-years-old groups was not significant according to the Mann-Whitney *U* test (*P* ​= ​0.295).

### 3.2 Correlations between urinary γGT and medical examination parameters

A statistically significant correlation was observed between u-γGT and age (r ​= ​−0.236, *P* ​= ​0.012), height (r ​= ​0.223, *P* ​= ​0.018), weight (r ​= ​0.208, *P* ​= ​0.027), u-Cre (r ​= ​0.892, *P* ​< ​0.001), u-Alb (r ​= ​0.432, *P* ​< ​0.001), and u-γGT/u-Cre (r ​= ​0.377, *P* ​< ​0.001). No significant correlations were observed between u-γGT and abdominal girth, systolic and diastolic blood pressures, HDL-C, LDL-C, TG, AST, ALT, s-γGT, HbA1c, s-Cre, eGFR, or u-Alb/u-Cre. No significant correlation was observed between u-γGT/u-Cre and medical examination parameters.

## 4 Discussion

In this study, we measured the u-γGT levels in (40–74)-year-old Japanese females (n ​= ​113). We also measured the creatinine-corrected value u-γGT/u-Cre, which is commonly used in clinics since the u-γGT of spot urine can be affected by the dilution and concentration of urine. A statistically significant positive correlation (*P* ​< ​0.001) was observed between u-γGT and u-Cre suggesting creatinine correction is reasonable for u-γGT. Conti et al. reported that correcting any tubular marker to u-Cre is not appropriate; however, u-Cre correction is appropriate for pure glomerulopathy [18]. This study differs from Conti et al. in the background of the participants, the type of urine collection, and the analytical method of creatinine. Study participant in the study of Conti et al. is a patient with renal disease on the other hand in our study, participants did not have renal disease. Urine sample in the study of Conti et al. was taken at 24 ​h urine, while in our study, urine type was spot urine. Analytical method for creatinine in the study of Conti et al. was Jaffe method, but we followed the enzymatic method. Therefore, we cannot compare both the studies directly, and further research is necessary to clarify the relationship between urine concentration and u-γGT. For the individuals in this study, u-γGT declined with age. We detected a statistically significant negative correlation between u-γGT and age (*P* ​= ​0.012), and a statistically significant difference in the u-γGT value between the (40–59)- and (60–74)-year-old groups (*P* ​= ​0.007). The decline of u-γGT with age has previously been described [12,19]. During the aging process, there is a decrease in the number of nephrons and a shortening of the renal tubular length [20,21]. Since u-γGT is of renal tubular epithelial cell origin, this result suggests that the decrease in renal tubular epithelial cells during aging influences u-γGT. By contrast, no such correlation with age was detected for u-γGT/Cre. In this study, a statistically significant correlation was observed between u-Cre and age (r ​= ​−0.237, *P* ​= ​0.012). The negative correlation between u-Cre and age is considered to reflect a decrease in the quantity of muscle with aging [13]. This is considered to reflect the fact that u-γGT and u-Cre decrease together during aging [12,19,22], and therefore their ratio will cancel out this association.

The u-Cre correction premise is that u-Cre excretion is 1 ​g/day. However, the quantity of u-Cre excretion changes under the influence of age, gender, race, body mass index, diabetes, and kidney function [22]. The u-Cre correction values in children and elderly women who have low u-Cre levels would be overestimated. If the quantity of u-Cre excretion is 1 ​g/day and the daily volume of urine of a Japanese woman is 1.2 ​L/day [23], the u-Cre concentration is estimated as 0.83 ​g/L. The medians of u-Cre for individuals in their 40s, 50s, 60s, and 70s in this study were 0.85, 0.74, 0.49, and 0.56 ​g/L, respectively. In this study, the urine samples were collected from 9:00 to 14:00. Concentrations of u-Cre are higher during the morning than in the evening [22,24]. In addition, eating and drinking are limited before specific health checkups. Collectively, this indicates that the average quantity of u-Cre excreted in the urine of elderly persons is ​< ​1 ​g/day. It is suggested that the u-γGT/u-Cre values were overestimated in elderly women. Namely, the expected decline of u-γGT with aging would be masked by the u-Cre correction. A suitable u-γGT correction method for spot urine in elderly persons is necessary. Recently, Jain [24] has reported a correction formula for u-Cre that is based on age, race/ethnicity, and gender, which may be a useful method. Urinary cystatin C concentration may be a better marker of renal tubular injury in renal-transplant recipients than u-γGT concentration [18]. For most urinary disease markers, including u-γGT, urinary dilution and concentration correction are required. Because there are many factors that may cause variations in urinary creatinine levels, it is difficult to evaluate the validity of creatinine correction values. Therefore, it is thought that urinary disease markers, such as urinary cystatin C, that do not require correction for urinary dilution and concentration are more useful.

With regards to physical parameters (height, weight, abdominal girth, systolic and diastolic blood pressure), a statistically significant correlation was observed between u-γGT and height or weight. Indeed, the number of nephrons has been reported to be proportional to body habitus [25,26], which may explain this correlation.

The u-γGT correlated with u-γGT/u-Cre and u-Alb. The correlation between u-γGT and u-γGT/u-Cre is quite natural because u-γGT/u-Cre was calculated from u-γGT. The correlation of u-γGT with u-Alb has also been reported in the younger population [19] and is known to fluctuate in response to urinary dilution and concentration.

This study has some limitations. First, the number of subjects (n = 30) in the 40–59-year-old age group was small and therefore, may not be good representative of the population from which they were drawn. The difference in the sample size between the (40–59)-year-old group (n = 30) and the 60-74-year-old group (n = 83) may reduce the statistical power of the Mann-Whitney *U* test. Second, there were only weak correlations between age and u-γGT or u-Cre. These observations might cast doubt on the suggestion of this study that creatinine-corrected u-γGT values are overestimated in elderly women.

In this study, we determined the urinary γGT distribution in (40–74)-year-old Japanese women receiving specific health checkups, whereby we aimed to provide basic data for the establishment of urinary γGT reference ranges for each included decade of age. Median u-γGT tended to decline with age, similar to previous studies [12,19]. For elderly women, the u-γGT/u-Cre level that is generally used as a disease biomarker may overestimate the amount of u-γGT excreted.

## Ethical considerations

The study plan was approved by the Ethical Committee for Medical and Health Research Involving Human Subjects of Chiba Institute of Science (approval number 28–24) and Department of Sports Science at Juntendo University (approval number 29–57). All the participants provided informed consent for the use of their urine samples and medical examination information. The Health and Welfare Center of Choshi-city (Chiba prefecture, Japan) implemented the organization of the medical examinations and provided permission for the use of the data in this study.

## CRediT authorship contribution statement

**Akihisa Hata:** Investigation, Formal analysis, Data curation, Writing - original draft, Visualization. **Maki Miyauchi:** Investigation, Resources. **Yoshio Suzuki:** Resources, Investigation, Project administration. **Yuki Otomo:** Investigation, Validation. **Noboru Fujitani:** Conceptualization, Writing - review & editing, Supervision.
